# Using CRISPR/Cas9 genome editing in human iPSCs for deciphering the pathogenicity of a novel *CCM1* transcription start site deletion

**DOI:** 10.3389/fmolb.2022.953048

**Published:** 2022-08-25

**Authors:** Robin A. Pilz, Dariush Skowronek, Motaz Hamed, Anja Weise, Elisabeth Mangold, Alexander Radbruch, Torsten Pietsch, Ute Felbor, Matthias Rath

**Affiliations:** ^1^ Department of Human Genetics, University Medicine Greifswald, and Interfaculty Institute of Genetics and Functional Genomics, University of Greifswald, Greifswald, Germany; ^2^ Department of Neurosurgery, University Hospital Bonn, Bonn, Germany; ^3^ Institute of Human Genetics, Jena University Hospital, Friedrich Schiller University, Jena, Germany; ^4^ Institute of Human Genetics, Medical Faculty and University Hospital Bonn, University of Bonn, Bonn, Germany; ^5^ Department of Neuroradiology, University Hospital Bonn, Bonn, Germany; ^6^ Institute of Neuropathology, DGNN Brain Tumor Reference Center, University of Bonn, Bonn, Germany

**Keywords:** cerebral cavernous malformation, CRISPR/Cas9, induced pluripotent stem cells, transcription start site, variant of unknown clinical significance

## Abstract

Cerebral cavernous malformations are clusters of aberrant vessels that can lead to severe neurological complications. Pathogenic loss-of-function variants in the *CCM1*, *CCM2*, or *CCM3* gene are associated with the autosomal dominant form of the disease. While interpretation of variants in protein-coding regions of the genes is relatively straightforward, functional analyses are often required to evaluate the impact of non-coding variants. Because of multiple alternatively spliced transcripts and different transcription start points, interpretation of variants in the 5′ untranslated and upstream regions of *CCM1* is particularly challenging. Here, we identified a novel deletion of the non-coding exon 1 of *CCM1* in a proband with multiple CCMs which was initially classified as a variant of unknown clinical significance. Using CRISPR/Cas9 genome editing in human iPSCs, we show that the deletion leads to loss of CCM1 protein and deregulation of *KLF2*, *THBS1*, *NOS3*, and *HEY2* expression in iPSC-derived endothelial cells. Based on these results, the variant could be reclassified as likely pathogenic. Taken together, variants in regulatory regions need to be considered in genetic CCM analyses. Our study also demonstrates that modeling variants of unknown clinical significance in an iPSC-based system can help to come to a final diagnosis.

## Introduction

Cerebral cavernous malformation (CCM) is a neurovascular disorder. Based on prospective, population-based studies in Scottish and American residents (Al-Shahi et al., 2003; Flemming et al., 2017) as well as a retrospective analysis of autopsies (Otten et al., 1989), it affects approximately one in 200 people. In CCM patients, mulberry-like vascular lesions can be visualized with appropriate magnetic resonance imaging (MRI) analyses in the brain or spinal cord (Akers et al., 2017). These lesions are characterized by irregular-structured and thin-walled endothelial channels that have an increased tendency to bleed. While many CCMs remain asymptomatic, hemorrhage from CCMs can lead to severe neurological deficits. Symptoms range from sensory and speech disturbances to seizures and stroke-like events even in young patients (Batra et al., 2009; Spiegler et al., 2014). Surgical excision and symptomatic treatment remain the only therapy options for patients to date.

Besides sporadic CCMs, 6%–7% of cases are due to autosomal dominant inherited heterozygous loss-of-function germline variants in the *CCM1* (*KRIT1*; OMIM: *604214), *CCM2* (*607929), or *CCM3* gene (*PDCD10*; *609118) (Spiegler et al., 2018b). Pathogenic variants in the *CCM1* gene are the most common cause of familial CCM disease (Spiegler et al., 2014). The diverse functions of CCM1 include Rap1- and HEG1-related stabilization of endothelial cell junctions (Glading et al., 2007; Gingras et al., 2012), regulation of DELTA-NOTCH, TGF-β and BMP6 signaling (Wüstehube et al., 2010; Maddaluno et al., 2013) as well as intracellular reactive oxygen species homeostasis (Goitre et al., 2010). Loss of CCM1 is also associated with altered KLF2/KLF4 (Zhou et al., 2016), THBS1 (Lopez-Ramirez et al., 2017), NOS3 (Lopez-Ramirez et al., 2021), and HEY2 (Wüstehube et al., 2010) protein or mRNA expression. Since *CCM1* was first described as a disease gene in 1999 (Laberge-le Couteulx et al., 1999; Sahoo et al., 1999), hundreds of *CCM1* mutations have been listed in international databases. Most of the known pathogenic variants are located in the coding region of the gene, with missense and frameshift variants accounting for the largest proportion. Variants in the non-coding region mainly alter the splicing process (Spiegler et al., 2018b; Ricci et al., 2021). Very little is known about variants in non-coding regions that potentially affect *CCM1* gene expression or protein function. From a general point of view, however, the 5′ and 3′ untranslated regions (UTRs) as well as promoter, enhancer or silencer motifs play an important role in disease development (Damjanovich et al., 2011; French and Edwards, 2020; Whiffin et al., 2020; Wright et al., 2021). Although potentially disease-causing, predicting the functional impact of non-coding variants is much more difficult than of protein-coding variants. As a result of clinical DNA sequencing, “variants of unknown clinical significance” (VUS) are detected in a significant proportion of patients (Rehm et al., 2015). Remarkably, a classification as VUS is more likely for variants outside the coding region, with ∼40% of coding variants versus ∼60% of all UTR variants classified as VUS in ClinVar (Ellingford et al., 2022).

With the guidelines published in 2015, the American College of Medical Genetics and Genomics (ACMG) and the Association for Molecular Pathology (AMP) established a standardized criteria-based system for interpreting sequence variants (Richards et al., 2015). Because the criteria have been designed for a broad application, further general and disease-specific modifications and refinements have emerged (Harrison et al., 2019). Apart from splicing variants, existing guidelines mainly focus on coding regions which makes it very challenging to apply the criteria to variants in UTRs of genes. Although genome interaction studies, quantitative trait locus mapping, and computational predictions can be useful for interpreting non-coding variants (Zhang and Lupski, 2015), functional assays may be necessary for a reliable assessment of the pathogenicity in many cases. The CRISPR/Cas9 technology is a powerful tool to engineer such variants in model organisms. Genome editing has also recently been applied to decipher VUS pathogenicity in human induced pluripotent stem cell (iPSC)-based models (Garg et al., 2018; Ma et al., 2018).

Here, we describe the accurate classification of a novel non-coding deletion in the *CCM1* gene following after CRISPR/Cas9 editing in human iPSCs.

## Materials and methods

### Genetic analyses and ethical considerations

Genetic analyses were performed with written informed consent according to the German Gene Diagnostics Act and with approval of the local ethics committee of the University Medicine Greifswald (No. BB 047/14). The NucleoSpin Blood L Kit (Macherey-Nagel, Düren, Germany) was used to isolate genomic DNA. Next-generation sequencing (NGS) gene panel analysis with the target region defined as all exons (±20 bp) of *CCM1* (Locus Reference Genomic sequence: LRG_650t1), *CCM2* (LRG_664t1,t2), and *CCM3* (LRG_651t1) was performed using a hybridization capture-based approach. For target enrichment and library preparation, an Agilent SureSelect^QXT^ custom enrichment kit (Panel ID: 3152261, Agilent Technologies, Santa Clara, United States) was used. The indexed library was sequenced on a MiSeq instrument (Illumina, San Diego, United States) with 2 × 150  bp paired-read runs. FASTQ file generation was done by the MiSeq Reporter Software (Illumina). Read mapping, alignment, and variant calling was performed by the SeqNext module of the Sequence Pilot v5.1.0 software (JSI Medical Systems, Ettenheim, Germany) that was also used for copy number variation (CNV) analyses in a read depth-based approach as described before (Much et al., 2019). To examine for translocation events, the generated FASTQ files were analyzed with the SureCall 4.2.1.10 software (Agilent Technologies). Polymerase chain reaction (PCR) was used to confirm the deletion of the *CCM1* exon 1. PCR products were separated by agarose gel electrophoresis. The band of interest was excised, purified with the Zymoclean Gel DNA Recovery Kit (Zymo Research, Irvine, United States), and analyzed by Sanger sequencing to determine the exact breakpoints of the deletion. All medical information and images presented here are published with written informed consent.

### Cell culture and reagents

HEK293T cells were cultured at 37°C and 5% CO_2_ in 1x Dulbecco’s Modified Eagle medium (DMEM) with high glucose (Thermo Fisher Scientific, Waltham, United States) and 10% fetal bovine serum (FBS; Thermo Fisher Scientific). AICS-0023 iPSCs (Allen Cell Collection, Coriell Institute, United States) were cultured at 37°C and 5% CO_2_ in Essential 8 Flex medium (Thermo Fisher Scientific) on plates coated with growth factor reduced matrigel (Corning, New York, United States) and passaged with 0.5 mM ethylenediaminetetraacetic acid (Thermo Fisher Scientific). Cell cultures were routinely checked for mycoplasma contamination by PCR. Oligonucleotides purchased from Integrated DNA Technologies and antibodies used in this study are listed in Supplementary Tables S1, S2.

### CRISPR/Cas9 editing, single-cell cloning, and karyotyping

To mimic the deletion identified in the index case, two crRNA:tracrRNA:Cas9 ribonucleoprotein (RNP) complexes were combined with Lipofectamine reagent in Opti-MEM I reduced serum medium (Thermo Fisher Scientific) for co-transfection of HEK293T or AICS-0023 cells. HEK293T cells were reverse transfected with RNP complexes as described before (Schwefel et al., 2020). For the AICS-0023 iPSC line, the crRNA:tracrRNA duplexes were complexed with Cas9 protein (Integrated DNA Technologies, Coralville, United States) in Opti-MEM I and formation of transfection complexes was performed in Opti-MEM I with Lipofectamine Stem Transfection Reagent (Thermo Fisher Scientific). After detaching with StemPro Accutase (Thermo Fisher Scientific), 130,000 cells were reverse transfected in Essential 8 medium (Thermo Fisher Scientific) supplemented with 10 µM Rho-associated protein kinase (ROCK) inhibitor Y-27632 (STEMCELL Technologies, Vancouver, Canada) on growth factor reduced matrigel-coated 24-well plates. After 1 day, the medium was replaced with Essential 8 Flex medium without ROCK inhibitor. Clonal *CCM1* ^del/del^ HEK293T and AICS-0023 iPSC lines were established by seeding genome-edited cells at a density of statistically 0.5 cells/well on 96-well plates in 1x DMEM and 10% FBS or on growth factor reduced matrigel-coated 96-well plates in Essential 8 Flex medium supplemented with CloneR (STEMCELL Technologies), respectively. Genomic DNA of clonally expanded cell lines was isolated with QuickExtract DNA Extraction Solution (Lucigen, Middleton, United States). Genotypes were determined by Sanger sequencing.

For generating control *CCM1* ^−/−^ AICS-0023 iPSC lines with frameshift variants in the coding region of *CCM1*, iPSCs were transfected with a single guide RNA (sgRNA):Cas9 RNP complex with a final RNP concentration of 30 nM. Genome editing efficiency was estimated after T7 endonuclease I (T7EI) digestion of annealed PCR amplicons as described before (Schwefel et al., 2020). After single-cell cloning, Sanger sequencing was used to determine the genotypes of the lines and to evaluate sequence changes at off-target sites predicted with the CHOPCHOP tool (Labun et al., 2019). Following standard procedures, chromosome analyses for *CCM1* ^−/−^ AICS-0023 iPSC clones were performed by GTG (G-bands by trypsin using Giemsa) staining of metaphase chromosomes.

### RT-qPCR, RT-PCR, and western blot

The Direct-zol RNA MiniPrep Plus Kit (Zymo Research) was used for purification of extracted RNA. Reverse transcription (RT) into cDNA was performed using the First Strand cDNA Synthesis Kit (Thermo Fisher Scientific). Transcript levels of *CCM1*, *ANKIB1*, *KLF2*, *KLF4*, *THBS1*, *NOS3*, and *HEY2* were quantified with SYBR Green-based quantitative PCR (qPCR) analyses performed on a Roche Light Cycler 480 instrument (Roche, Basel, Switzerland) or on a QuantStudio 7 Flex Real-Time PCR System (Applied Bioystems, Waltham, United States). *RPLP0* served as an endogenous control. In RT-PCR, 10 ng of transcribed cDNA were amplified in 28 (*RPLP0*) or 33 cycles (*CCM1* and *ANKIB1*). The GraphPad prism software was used for data analysis (GraphPad Software, San Diego, United States).

For western blot analyses, total protein extracted with RIPA Lysis and Extraction Buffer (Thermo Fisher Scientific) was separated on a 10% TGX Stain-Free FastCast sodium dodecyl sulfate-polyacrylamide gel (Bio-Rad, Hercules, United States) and subsequently transferred to a polyvinylidene fluoride membrane. The iBind Flex Western System (Thermo Fisher Scientific) was used for immunostaining according to the manufacturer’s instructions. Stripping of the membrane was performed with ROTI Free Stripping Buffer 2.2 plus (Carl Roth, Karlsruhe, Germany). Blot documentation of Stain-Free total protein and chemiluminometric signal detection was performed using a ChemiDoc XRS+ imager. To determine relative CCM1 protein expression, normalized band intensities were calculated with the ImageLab software (v5.2.1, Bio-Rad). GAPDH or total protein was used as a loading control and volume intensities of the detected protein bands were normalized to the volume intensities of the corresponding GAPDH bands or total protein fraction.

### Differentiation procedures and immunofluorescent staining

Cells were fixed with 4% paraformaldehyde for 15–20 min. IPSCs were stained for pluripotency markers OCT4, SSEA4, SOX2, and TRA-1-60 using the PSC 4-Marker Immunocytochemistry Kit (Thermo Fisher Scientific) with alternative secondary antibodies for SSEA4 and SOX2 staining. Differentiation of iPSCs into endothelial cells (ECs) was performed in 6-well-plates using the STEMdiff Endothelial Differentiation Kit (STEMCELL Technologies). Directed differentiation of iPSCs into all three germ layers was performed in 24-well-plates using the STEMdiff Trilineage Differentiation Kit (STEMCELL Technologies). The Immunofluorescence Application Solutions Kit (Cell Signaling, Danvers, United States) was used for staining of CD31, VE-Cadherin, PAX6, TUJ-1, and α-SMA. For markers Brachyury, SOX17, and FOXA2, cells were permeabilized and blocked in 0.3% Triton X-100, 1% bovine serum albumin, and 10% normal donkey serum for 45 min. Cells were incubated with primary antibodies overnight at 4°C and with secondary antibodies for 60 min at room temperature in the dark. Nuclei were stained with DAPI or Hoechst 33342 (Thermo Fisher Scientific).

## Results

### Clinical findings and genetic analyses

**FIGURE 1:**
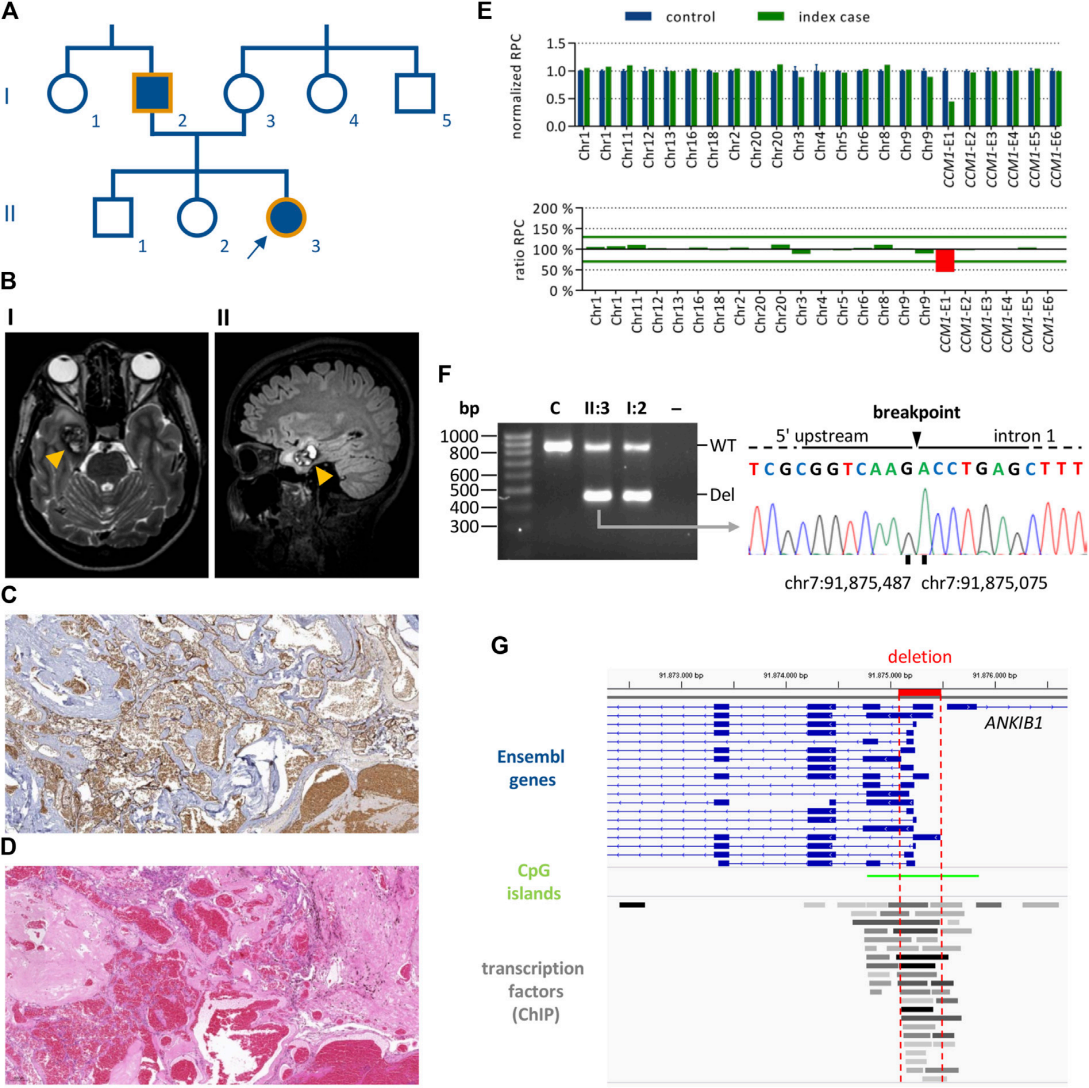
Identification of a *CCM1* exon 1 deletion in a proband with multiple CCMs. **(A)** Pedigree of the CCM index case (II:3, arrow). Filled forms indicate individuals with CCM-associated symptoms. Orange outlines indicate family members for whom genetic analyses have been performed. **(B)** Magnetic resonance imaging for the index case showing a right temporo-mesial CCM (orange arrow heads). I: T2-weighted sequence, II: FLAIR sequence. **(C,D)** Histological analyses of the CCM shown in B (×5 magnification). **(C)** CD34 staining, **(D)** HE staining. **(E)** NGS-based CNV analyses for the index case showing a heterozygous deletion of *CCM1* exon 1. **(F)** PCR amplification of the deletion region for index case II:3 and her father I:2 (WT, wild-type band; Del, deletion band; C, healthy control; –, negative control). The deletion breakpoints were determined by Sanger sequencing of the deletion band. **(G)** The deletion (highlighted in red) affects all relevant Ensembl transcripts of *CCM1*. The location of CpG islands and transcription factor binding sites are shown (ChIP, chromatin immunoprecipitation).

The female index case II:3 (Figure 1A) first presented in our outpatient clinic at the age of 24 with headaches and bilateral dysesthesias of the toes. Brain MRI analysis revealed a cavernoma in her right medial temporal lobe (Figure 1B) which was resected because of size progression and perifocal edema. Numerous malformed vessels, often with very severe fibrous wall thickening, and signs of recurrent bleeding events were observed in the histological analyses of the CCM tissue (Figures 1C,D). In addition to the temporo-mesial cavernoma, multiple small CCMs were identified in the left parieto-occipital lobe, the left periventricular region, the cerebellar vermis, and the head of the caudate nucleus. MRI analysis of the patient’s father (I:2) also revealed hemosiderin deposits consistent with previous hemorrhages of small CCMs. Although there were no other symptomatic family members, the personal and family history of the index case suggested familial CCM disease.

NGS-based gene panel analysis did not reveal a pathogenic single nucleotide (SNV) or small indel variant but a high number of split reads in intron 1 of *CCM1*. In line with these data, NGS-based CNV detection with the SeqNext module of the Sequence Pilot software and the Agilent SureCall tool indicated a heterozygous deletion of *CCM1* exon 1 (Figure 1E). PCR amplification of the deletion region resulted in a wild-type band and an approximately 400 base pair (bp) shorter band for the index case and her father. The exact breakpoints of the 411 bp deletion were determined by Sanger sequencing (NC_000007.13:g.91875486_91875076del; Figure 1F). As shown in the Integrative Genomics Viewer (Robinson et al., 2011), the identified deletion includes the transcription start sites (TSS) of all relevant *CCM1* transcripts listed in the Ensembl database and covers several transcription factor binding sites (Figure 1G). Following the ACMG standards and guidelines for the interpretation of sequence variants (Richards et al., 2015), the deletion was classified as VUS (criteria PM1 and PP4). Notably, the PVS1 criterion was not applied because the deletion neither affects the reading frame nor canonical splice sites of the coding exons of *CCM1*.

### Modeling a *CCM1* knockout in an iPSC-based cell culture system

**FIGURE 2:**
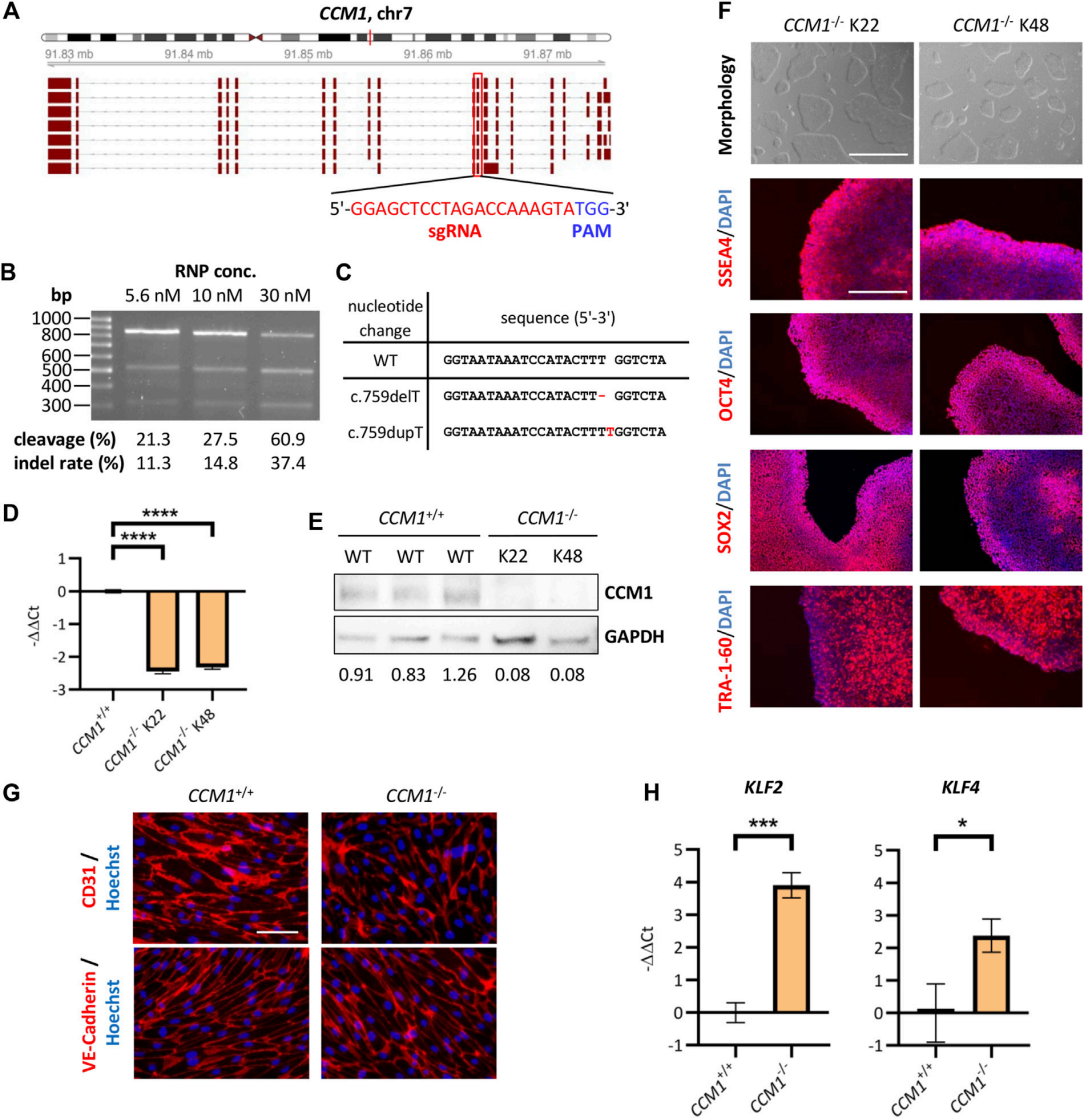
Establishment and validation of an iPSC-based *CCM1* knockout cell culture model. **(A)** Schematic depiction of the exon-intron structure of the *CCM1* gene and its relevant transcripts. The CRISPR/Cas9 target region of the sgRNA (red box) is part of all functional transcripts. **(B)** Optimization of the CRISPR/Cas9 RNP transfection showed best cleavage efficiency in the T7EI assay when using a final RNP concentration of 30 nM. **(C)** DNA sequences of the AICS-0023-derived *CCM1* ^−/−^ clones shown as a sequence alignment. **(D)** RT-qPCR revealed a marked reduction of *CCM1* mRNA expression in knockout lines (*CCM1* ^+/+^: *n* = 3, *CCM1* ^−/−^ K22/K48: *n* = 3 each). **(E)** Western blot analyses of clonal lines verified absence of CCM1 protein in *CCM1* ^−/−^ cells. Expression levels normalized to the wild-type (WT) control group are given. **(F)** Images of the morphology (scale = 1000 µm) and immunofluorescence analyses for stem cell markers SSEA4, OCT4, SOX2, and TRA-1-60 (scale = 400 µm). **(G)** AICS-0023-derived ECs expressed markers CD31 and VE-Cadherin. Representative immunofluorescent images are shown (scale = 75 µm). **(H)** AICS-0023-derived *CCM1* ^−/−^ ECs showed increased *KLF2* and *KLF4* mRNA expression (*CCM1* ^+/+^: *n* = 3, *CCM1* ^−/−^: *n* = 3). Data are presented as mean and standard deviation (SD). Two-tailed unpaired Student’s *t* test **(D, H)** was used for statistical analyses: *p < 0.05, ***p < 0.001, ****p < 0.0001.

To come to a final molecular diagnosis in our family, we decided to use CRISPR/Cas9 genome editing to rebuild the variant in an iPSC-based *in vitro* system. As a positive control for functional and molecular assays, we first generated *CCM1* ^−/−^ AICS-0023 iPSCs with bona fide loss-of-function variants on both alleles, mimicking the second-hit inactivation in heterozygous mutation carriers. Somatic inactivation of the remaining wild-type allele in ECs by a second mutation is described as a critical step in CCM formation (Pagenstecher et al., 2009; McDonald et al., 2014). The sgRNA target sequence used in our approach was located in exon 10, which is part of all functional *CCM1* transcripts (Figure 2A). We optimized the genome editing protocol using different sgRNA:Cas9 RNP concentrations and observed the highest indel rate of 37.4% at a concentration of 30 nM (Figure 2B). Clonal *CCM1* ^−/−^ iPSCs generated by limiting dilution cloning were checked for CRISPR/Cas9-induced loss-of-function variants in the target region by Sanger sequencing and NGS. Hence, we established one iPSC line with two compound heterozygous frameshift variants and one line with a homozygous 1 bp duplication (Figure 2C, Supplementary Figure S1). No sequence changes were observed at seven top off-target sites (Supplementary Figure S2). RT-qPCR and western blot analyses confirmed functional *CCM1* knockout (Figures 2D,E, Supplementary Figure S3). The *CCM1* ^−/−^ iPSC lines displayed a typical morphology, had a normal male karyotype (46, XY), and expressed the pluripotency markers SSEA4, OCT4, SOX2, and TRA-1-60 (Figure 2F, Supplementary Figure S1). Furthermore, they could be differentiated into ecto-, meso- and endoderm (Supplementary Figure S1).

As *CCM1* inactivation is known to induce characteristic gene expression changes in ECs, we generated iPSC-derived ECs and verified the expression of the endothelial markers CD31 and VE-Cadherin (Figure 2G). As expected, mRNA expression of *KLF2* and *KLF4* was significantly increased in iPSC-derived *CCM1* ^−/−^ ECs (Figure 2H). In summary, we have hereby established an iPSC-based *CCM1* knockout model that served as a benchmark system in assessing the pathogenicity of the identified *CCM1* TSS deletion.

### Using CRISPR/Cas9 editing to mimic the identified deletion in HEK293T cells

**FIGURE 3:**
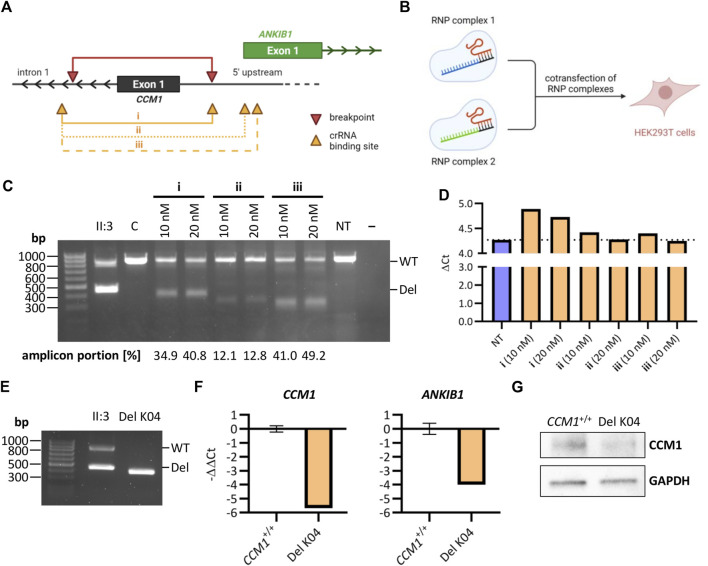
Reconstructing the identified deletion with CRISPR/Cas9 genome editing in HEK293T cells. **(A)** Schematic representation of the identified deletion in *CCM1* (red) and crRNA binding sites (orange) (Image was created with BioRender.com). The crRNA combinations i, ii, and iii were used to generate a deletion similar to the identified variant in the index case. **(B)** HEK293T cells were co-transfected with two RNP complexes for mimicking the deletion (Image was created with BioRender.com). **(C)** PCR amplification of the deletion region after co-transfection of HEK293T cells with different combinations of crRNAs and at different final RNP complex concentrations. **(D)** *CCM1* RT-qPCR for different HEK293T cell mixtures after transfection (*n* = 1 each, NT = not transfected). **(E)** PCR amplification of the deletion region for a homozygous HEK293T cell clone after using crRNA combination i for transfection. **(F)** RT-qPCR for HEK293T clone Del K04 shows a clear downregulation of *CCM1* and *ANKIB1* mRNA (*CCM1* ^+/+^: *n* = 3, Del K04: *n* = 1). **(G)** Reduction of CCM1 protein expression for HEK293T clone Del K04 is shown by western blot. Data are presented as mean and standard deviation (SD).

We next used easy-to-transfect and highly proliferative HEK293T cells to test the genome editing efficiencies of three crRNA combinations with binding sites near the breakpoints of the identified deletion (Figure 3A). For each combination, HEK293T cells were co-transfected with two RNP complexes at a final concentration of 10 nM or 20 nM (Figure 3B). PCR analyses indicated high efficiencies for the combinations i and iii (Figure 3C). RT-qPCR demonstrated decreased *CCM1* mRNA expression in HEK293T cells transfected with crRNA combination i (Figure 3D). The deletion induced by this combination most accurately reflects the variant identified in index case II:3 (Figure 3A). Therefore, we next established a homozygous cell clone (Del K04) from this cell mixture (Figure 3E) and verified reduced *CCM1* mRNA and protein expression in this HEK293T clone (Figures 3F,G, Supplementary Figure S3). Interestingly, we also found reduced mRNA expression of the *ANKIB1* gene, whose TSS is close to the deletion region (Figures 3A,F).

### Gene expression analyses in iPSC-derived endothelial cells support pathogenicity of the identified deletion

**FIGURE 4:**
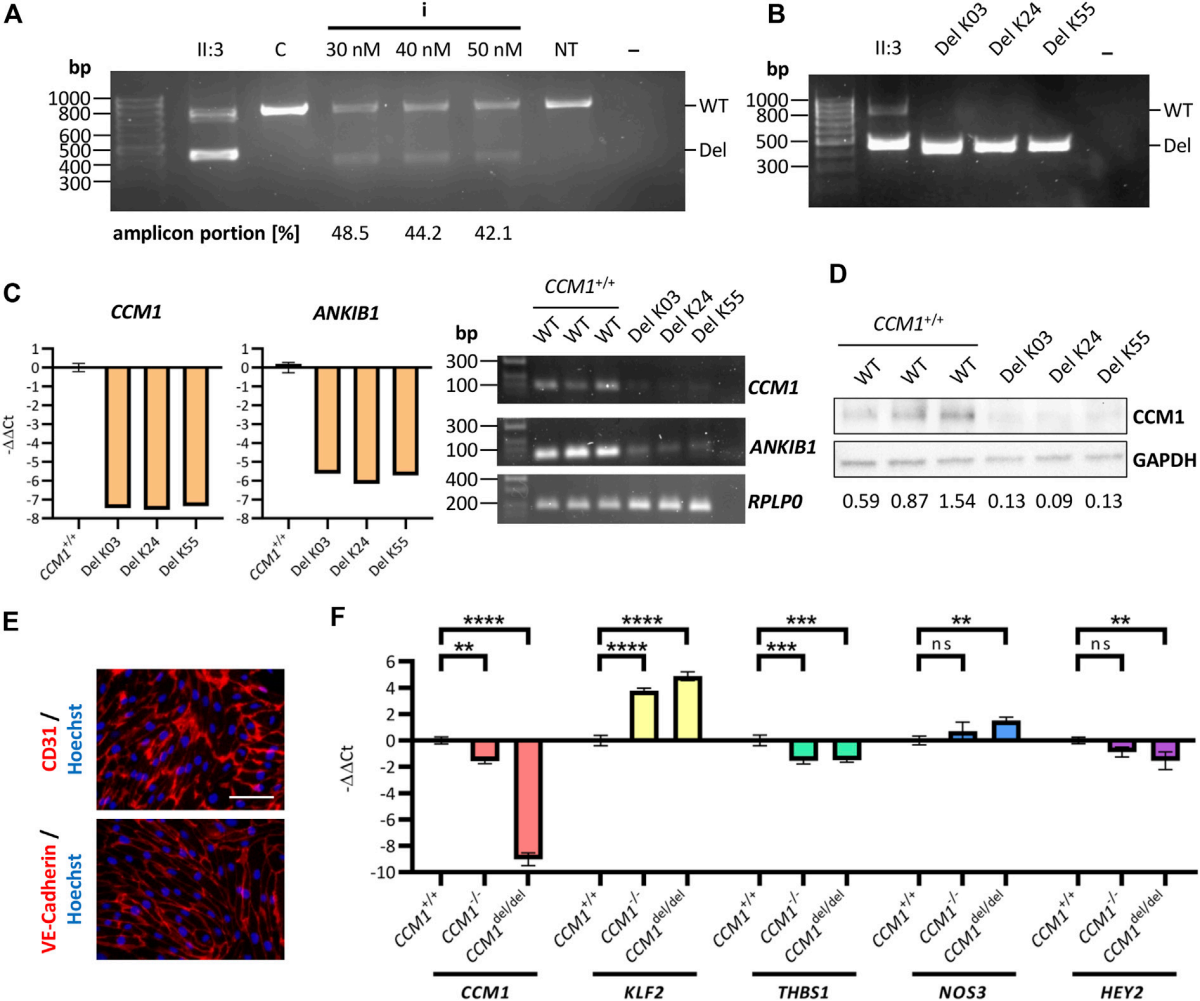
Analyzing the functional impact of the identified deletion in an iPSC-based cell culture model. **(A)** PCR amplification of the deletion region after co-transfection (crRNA combination i, Figure 3A) of AICS-0023 iPSCs at different final RNP complex concentrations. **(B)** PCR amplification of the deletion region for three AICS-0023 clones with a biallelic CRISPR/Cas9 induced deletion. **(C)** Marked reduction of *CCM1* and *ANKIB1* mRNA in *CCM1* ^del/del^ iPSC clones shown by RT-qPCR (left panels; *CCM1* ^+/+^: *n* = 3, Del K03/K24/K55: *n* = 1 each) and RT-PCR (right panel). **(D)** Western blot analysis show loss of CCM1 protein in *CCM1* ^del/del^ clones. Expression levels normalized to the wild-type (WT) control group are given. **(E)** IPSC-derived *CCM1* ^del/del^ ECs express markers CD31 and VE-Cadherin. Representative immunofluorescence images are shown (scale = 75 µm). **(F)** *CCM1, KLF2*, *THBS1*, *NOS3*, and *HEY2* RT-qPCR analyses for differentiated *CCM1* ^+/+^, *CCM1* ^−/−^, and *CCM1* ^del/del^ ECs. Data are presented as mean and standard deviation (SD). One-way ANOVA with multiple comparisons test **(F)** was used for statistical analyses: **p < 0.01, ***p < 0.001, ****p < 0.0001.

Having determined the optimal crRNA combination, we finally generated AICS-0023 iPSCs with the *CCM1* TSS deletion on both alleles (*CCM1* ^del/del^). Increasing the final RNP concentration did not improve editing efficiency (Figure 4A). By single-cell cloning, three *CCM1* ^del/del^ iPSC lines could be established (Del K03, Del K24, Del K55) (Figure 4B). Consistent with the hypothesis that *CCM1* TSS deletion is pathogenic, a significantly reduced *CCM1* expression was shown by RT-qPCR and western blot analyses, respectively (Figures 4C,D, Supplementary Figure S3). *ANKIB1* mRNA expression was also reduced in *CCM1* ^del/del^ iPSCs (Figure 4C). We then differentiated the *CCM1* ^del/del^ iPSCs into ECs (Figure 4E). Interestingly, complete loss of *CCM1* mRNA expression was observed for *CCM1* ^del/del^ ECs, whereas it was greatly reduced but still existent in *CCM1* ^−/−^ ECs (Figure 4F). A similar effect was observed for *KLF2*, *NOS3,* and *HEY2*, with the more pronounced dysregulation in *CCM1* ^del/del^ ECs (Figure 4F). We also analyzed *THBS1* expression and found equal reduction in *CCM1* ^−/−^ and *CCM1* ^del/del^ ECs (Figure 4F).

Taken together, the functional *in vitro* studies presented here provide strong evidence for the pathogenicity of the identified *CCM1* TSS deletion. Following the ACMG guideline, it can now be classified as likely pathogenic (PS3, PM1, and PP4).

## Discussion

Coding variants account for the majority of pathogenic variants in the *CCM* genes. However, with the novel *CCM1* TSS deletion characterized in our study, we demonstrate that non-coding variants also need to be considered and that CRISPR/Cas9 editing in iPSCs can help with the interpretation of VUS.

Because mutational hotspots cannot be determined, genetic testing for CCM typically involves analysis of all coding exons and exon-intron boundaries of *CCM1*, *CCM2*, and *CCM3*. NGS gene panel analysis has proven superior to a stepwise approach in this context (Spiegler et al., 2018b). It allows parallel screening for SNVs, indels, and CNVs (Much et al., 2019). NGS-based CNV analyses are particularly advantageous if regions of interest such as non-coding exons are not covered by commercial multiplex ligation-dependent probe amplification kits. Nonetheless, no causative variant is identified in 2%–13% of familial CCM cases by current genetic analyses (Spiegler et al., 2018b). Apart from structural variants (Spiegler et al., 2018a; Pilz et al., 2020) and somatic mosaicism (McDonald et al., 2014), the here identified *CCM1* TSS deletion highlights that variants in regulatory regions that are not always analyzed in routine diagnostics may account for part of the missing heritability in CCM disease.

However, the clinical interpretation of variants in 5′ UTR or promoter regions is challenging. In the absence of experimental data, the identified *CCM1* TSS deletion would have been classified as VUS using the widely accepted ACMG guidelines. As the coding region of *CCM1* starts only in exon 5 (LRG_650t1), the deletion does not affect the reading frame but results in loss of the TSS. In the gnomAD structural variant database (Collins et al., 2020) with its 10,847 genome data sets no deletion only covering exon 1 is registered, but information from larger cohorts on the variant’s frequency in the general population is still limited. Deletions of non-coding *CCM1* exons described in the literature so far are also considerably larger than the variant described here (Riant et al., 2013; Mondéjar et al., 2014). Furthermore, the occurrence of transcripts with alternative TSSs that may rescue the phenotype could not be excluded. Interestingly, 5′ RACE analysis revealed different TSSs and alternative splicings of the *CCM1* 5′ UTR. Intragenic TSSs were shown by quantitative transcription studies and detection of five promoter sequences by MPromDB analysis in the 5′ UTR and intragenic region of *CCM1* (Mondéjar et al., 2016). Even with consideration of further relevant recommendations for interpreting the loss-of-function variants (Abou Tayoun et al., 2018) and adaptations for single-gene copy number variants (Brandt et al., 2020) the PVS1 criterion could not be used in this case. Yet, the distinction between a pathogenic and benign variant is of great importance for accurate diagnosis, appropriate clinical management and genetic counselling of family members.

According to the ACMG guidelines, well-established *in vitro* analyses can be strong evidence of a variant’s pathogenic or benign impact. Recently published recommendations aiming for a consistent clinical interpretation of non-coding variants have also highlighted the importance of functional evidence (Ellingford et al., 2022). In this context, iPSC-based disease modeling, which has developed rapidly in recent years, can be very helpful. The ability of iPSCs to differentiate into all cell types and their unlimited availability gives them an enormous advantage over primary and immortalized cell lines (Grskovic et al., 2011). Benchmarking and the use of appropriate controls, however, is a critical aspect of well-designed *in vitro* analyses to establish the range of the assay readout and to define thresholds (Brnich et al., 2019). The introduction of genetic variants into iPSCs with CRISPR/Cas9 genome editing is particularly valuable because it allows generation of isogenic lines, thereby reducing variability due to genetic background. We therefore used CRISPR/Cas9 genome editing in human iPSCs to provide functional evidence for the pathogenicity of the novel *CCM1* TSS deletion identified in our index case. The almost complete loss of *CCM1* gene expression in *CCM1* ^del/del^ cells was strong evidence for the pathogenicity of the variant and sufficient to classify this deletion as likely pathogenic following the ACMG guidelines. Thus, we were able to confirm the molecular CCM diagnosis for the family and can now offer genetic analysis to further at-risk relatives. We also demonstrated a reduced expression of *ANKIB1* in *CCM1* ^del/del^ iPSCs. However, no association can currently be established between decreased *ANKIB1* expression and the clinical phenotype of the index patient since little is known about the function of ANKIB1 so far.

Interestingly, our study led to another, rather unexpected, finding. While *CCM1* transcript levels were extremely low and thus hardly detectable in *CCM1* ^del/del^ iPSCs, residual *CCM1* transcript was still present in *CCM1* ^−/−^ knockout iPSCs with biallelic frameshift variants in the coding region of *CCM1*. We decided to study this effect in more detail and differentiated *CCM1* ^del/del^ and *CCM1* ^−/−^ iPSCs to ECs. While a very well-known consequence of *CCM1* inactivation in ECs, namely upregulation of *KLF2* (Zhou et al., 2016), was observed in both cell types, it was more pronounced in iPSC-derived *CCM1* ^del/del^ ECs. A similar effect was found for the expression of *NOS3* and *HEY2*. Only *THBS1* expression was equally reduced in iPSC-derived *CCM1* ^del/del^ and *CCM1* ^−/−^ ECs. It has been shown in studies with different animal models that degradation of mutant mRNA is a possible trigger of genetic compensation mechanisms that may account for phenotypic differences between stable mutants and transient knockdowns (Rossi et al., 2015; El-Brolosy et al., 2019). This mechanism might also be an explanation for the more pronounced molecular consequences of the *CCM1* TSS deletion that blocks *CCM1* expression already at the transcriptional level. Thus, our *in vitro* analyses demonstrate the critical regulatory function of the region affected by the deletion. However, we were not able to directly compare homozygous *CCM1* ^del/del^ with heterozygous *CCM1* ^WT/del^ ECs, since we could not establish *CCM1* ^WT/del^ iPSCs. Further, possible off-target effects in iPSC-derived *CCM1* ^del/del^ ECs cannot be completely excluded. Yet, *CCM1* is a well-characterized disease gene and the observed molecular effects in *CCM1* ^del/del^ ECs correlate very well with the results from established *CCM1* knockout models.

In conclusion, our study expands the *CCM* mutation spectrum and illustrates that non-coding variants may be a cause of disease in apparently mutation-negative CCM cases. Moreover, we demonstrate that CRISPR/Cas9 editing in iPSCs represents a powerful approach for variant interpretation and can provide a promising platform for basic research or therapeutic CCM studies. Using iPSC-derived human brain microvascular endothelial-like cells and mosaic vascular organoids, we were recently able to show abnormal proliferation of *CCM3* mutant ECs in co-culture with wild-type ECs (Rath et al., 2022). In the future, novel patient-specific, co-culture or three-dimensional iPSC-based cell culture models could give further insight into CCM pathogenesis.

## Data Availability

The original contributions presented in the study are included in the article/Supplementary Material, further inquiries can be directed to the corresponding author.

## References

[B1] Abou Tayoun A. N. Pesaran T. DiStefano M. T. Oza A. Rehm H. L. Biesecker L. G. (2018). Recommendations for interpreting the loss of function PVS1 ACMG/AMP variant criterion. Hum. Mutat. 39 (11), 1517–1524. 10.1002/humu.23626 30192042 PMC6185798

[B2] Akers A. Al-Shahi Salman R. Awad I. A. Dahlem K. Flemming K. Hart B. (2017). Synopsis of guidelines for the clinical management of cerebral cavernous malformations: Consensus recommendations based on systematic literature review by the angioma alliance scientific advisory board clinical experts panel. Neurosurgery 80 (5), 665–680. 10.1093/neuros/nyx091 28387823 PMC5808153

[B3] Al-Shahi R. Bhattacharya J. J. Currie D. G. Papanastassiou V. Ritchie V. Roberts R. C. (2003). Prospective, population-based detection of intracranial vascular malformations in adults: the scottish intracranial vascular malformation study (SIVMS). Stroke 34 (5), 1163–1169. 10.1161/01.STR.0000069018.90456.C9 12702837

[B4] Batra S. Lin D. Recinos P. F. Zhang J. Rigamonti D. (2009). Cavernous malformations: natural history, diagnosis and treatment. Nat. Rev. Neurol. 5 (12), 659–670. 10.1038/nrneurol.2009.177 19953116

[B5] Brandt T. Sack L. M. Arjona D. Tan D. Mei H. Cui H. (2020). Adapting ACMG/AMP sequence variant classification guidelines for single-gene copy number variants. Genet. Med. 22 (2), 336–344. 10.1038/s41436-019-0655-2 31534211

[B6] Brnich S. E. Abou Tayoun A. N. Couch F. J. Cutting G. R. Greenblatt M. S. Heinen C. D. (2019). Recommendations for application of the functional evidence PS3/BS3 criterion using the ACMG/AMP sequence variant interpretation framework. Genome Med. 12 (1), 3. 10.1186/s13073-019-0690-2 31892348 PMC6938631

[B7] Collins R. L. Brand H. Karczewski K. J. Zhao X. Alföldi J. Francioli L. C. (2020). A structural variation reference for medical and population genetics. Nature 581 (7809), 444–451. 10.1038/s41586-020-2287-8 32461652 PMC7334194

[B8] Damjanovich K. Langa C. Blanco F. J. McDonald J. Botella L. M. Bernabeu C. (2011). 5'UTR mutations of *ENG* cause hereditary hemorrhagic telangiectasia. Orphanet J. Rare Dis. 6, 85. 10.1186/1750-1172-6-85 22192717 PMC3277489

[B9] El-Brolosy M. A. Kontarakis Z. Rossi A. Kuenne C. Günther S. Fukuda N. (2019). Genetic compensation triggered by mutant mRNA degradation. Nature 568 (7751), 193–197. 10.1038/s41586-019-1064-z 30944477 PMC6707827

[B10] Ellingford J. M. Ahn J. W. Bagnall R. D. Baralle D. Barton S. Campbell C. (2022). Recommendations for clinical interpretation of variants found in non-coding regions of the genome. Genome Med. 14 (1), 73. 10.1186/s13073-022-01073-3 35850704 PMC9295495

[B11] Flemming K. D. Graff-Radford J. Aakre J. Kantarci K. Lanzino G. Brown R. D. Jr. (2017). Population-based prevalence of cerebral cavernous malformations in older adults: mayo clinic study of aging. JAMA Neurol. 74 (7), 801–805. 10.1001/jamaneurol.2017.0439 28492932 PMC5647645

[B12] French J. D. Edwards S. L. (2020). The role of noncoding variants in heritable disease. Trends Genet. 36 (11), 880–891. 10.1016/j.tig.2020.07.004 32741549

[B13] Garg P. Oikonomopoulos A. Chen H. Li Y. Lam C. K. Sallam K. (2018). Genome editing of induced pluripotent stem cells to decipher cardiac channelopathy variant. J. Am. Coll. Cardiol. 72 (1), 62–75. 10.1016/j.jacc.2018.04.041 29957233 PMC6050025

[B14] Gingras A. R. Liu J. J. Ginsberg M. H. (2012). Structural basis of the junctional anchorage of the cerebral cavernous malformations complex. J. Cell Biol. 199 (1), 39–48. 10.1083/jcb.201205109 23007647 PMC3461514

[B15] Glading A. Han J. Stockton R. A. Ginsberg M. H. (2007). KRIT-1/CCM1 is a Rap1 effector that regulates endothelial cell cell junctions. J. Cell Biol. 179 (2), 247–254. 10.1083/jcb.200705175 17954608 PMC2064761

[B16] Goitre L. Balzac F. Degani S. Degan P. Marchi S. Pinton P. (2010). KRIT1 regulates the homeostasis of intracellular reactive oxygen species. PLoS One 5 (7), e11786. 10.1371/journal.pone.0011786 20668652 PMC2910502

[B17] Grskovic M. Javaherian A. Strulovici B. Daley G. Q. (2011). Induced pluripotent stem cells-opportunities for disease modelling and drug discovery. Nat. Rev. Drug Discov. 10 (12), 915–929. 10.1038/nrd3577 22076509

[B18] Harrison S. M. Biesecker L. G. Rehm H. L. (2019). Overview of specifications to the ACMG/AMP variant interpretation guidelines. Curr. Protoc. Hum. Genet. 103 (1), e93. 10.1002/cphg.93 31479589 PMC6885382

[B19] Laberge-le Couteulx S. Jung H. H. Labauge P. Houtteville J. P. Lescoat C. Cecillon M. (1999). Truncating mutations in *CCM1*, encoding KRIT1, cause hereditary cavernous angiomas. Nat. Genet. 23 (2), 189–193. 10.1038/13815 10508515

[B20] Labun K. Montague T. G. Krause M. Torres Cleuren Y. N. Tjeldnes H. Valen E. (2019). CHOPCHOP v3: expanding the CRISPR web toolbox beyond genome editing. Nucleic Acids Res. 47 (W1), W171–W174. 10.1093/nar/gkz365 31106371 PMC6602426

[B21] Lopez-Ramirez M. A. Fonseca G. Zeineddine H. A. Girard R. Moore T. Pham A. (2017). Thrombospondin1 (TSP1) replacement prevents cerebral cavernous malformations. J. Exp. Med. 214 (11), 3331–3346. 10.1084/jem.20171178 28970240 PMC5679163

[B22] Lopez-Ramirez M. A. Lai C. C. Soliman S. I. Hale P. Pham A. Estrada E. J. (2021). Astrocytes propel neurovascular dysfunction during cerebral cavernous malformation lesion formation. J. Clin. Invest. 131 (13), 139570. 10.1172/JCI139570 34043589 PMC8245174

[B23] Ma N. Zhang J. Z. Itzhaki I. Zhang S. L. Chen H. Haddad F. (2018). Determining the pathogenicity of a genomic variant of uncertain significance using CRISPR/Cas9 and human-induced pluripotent stem cells. Circulation 138 (23), 2666–2681. 10.1161/CIRCULATIONAHA.117.032273 29914921 PMC6298866

[B24] Maddaluno L. Rudini N. Cuttano R. Bravi L. Giampietro C. Corada M. (2013). EndMT contributes to the onset and progression of cerebral cavernous malformations. Nature 498 (7455), 492–496. 10.1038/nature12207 23748444

[B25] McDonald D. A. Shi C. Shenkar R. Gallione C. J. Akers A. L. Li S. (2014). Lesions from patients with sporadic cerebral cavernous malformations harbor somatic mutations in the CCM genes: evidence for a common biochemical pathway for CCM pathogenesis. Hum. Mol. Genet. 23 (16), 4357–4370. 10.1093/hmg/ddu153 24698976 PMC4103679

[B26] Mondéjar R. Solano F. Rubio R. Delgado M. Pérez-Sempere A. González-Meneses A. (2014). Mutation prevalence of cerebral cavernous malformation genes in Spanish patients. PLoS One 9 (1), e86286. 10.1371/journal.pone.0086286 24466005 PMC3900513

[B27] Mondéjar R. Delgado M. Solano F. Izquierdo G. Martinez-Mir A. Lucas M. (2016). Analysis of *CCM1* expression uncovers novel minor-form exons and variable splicing patterns. Genes Genomics 38 (9), 879–889. 10.1007/s13258-016-0435-1

[B28] Much C. D. Schwefel K. Skowronek D. Shoubash L. von Podewils F. Elbracht M. (2019). Novel pathogenic variants in a cassette exon of *CCM2* in patients with cerebral cavernous malformations. Front. Neurol. 10, 1219. 10.3389/fneur.2019.01219 31824402 PMC6879547

[B29] Otten P. Pizzolato G. P. Rilliet B. Berney J. (1989). 131 cases of cavernous angioma (cavernomas) of the CNS, discovered by retrospective analysis of 24, 535 autopsies (in French). Neurochirurgie 35 (2), 82128–83131. 2674753

[B30] Pagenstecher A. Stahl S. Sure U. Felbor U. (2009). A two-hit mechanism causes cerebral cavernous malformations: complete inactivation of CCM1, CCM2 or CCM3 in affected endothelial cells. Hum. Mol. Genet. 18 (5), 911–918. 10.1093/hmg/ddn420 19088124 PMC2640205

[B31] Pilz R. A. Schwefel K. Weise A. Liehr T. Demmer P. Spuler A. (2020). First interchromosomal insertion in a patient with cerebral and spinal cavernous malformations. Sci. Rep. 10 (1), 6306. 10.1038/s41598-020-63337-5 32286434 PMC7156631

[B32] Rath M. Schwefel K. Malinverno M. Skowronek D. Leopoldi A. Pilz R. A. (2022). Contact-dependent signaling triggers tumor-like proliferation of *CCM3* knockout endothelial cells in co-culture with wild-type cells. Cell. Mol. Life Sci. 79 (6), 340. 10.1007/s00018-022-04355-6 35661927 PMC9166869

[B33] Rehm H. L. Berg J. S. Brooks L. D. Bustamante C. D. Evans J. P. Landrum M. J. (2015). ClinGen- the clinical genome resource. N. Engl. J. Med. 372 (23), 2235–2242. 10.1056/NEJMsr1406261 26014595 PMC4474187

[B34] Riant F. Cecillon M. Saugier-Veber P. Tournier-Lasserve E. (2013). CCM molecular screening in a diagnosis context: novel unclassified variants leading to abnormal splicing and importance of large deletions. Neurogenetics 14 (2), 133–141. 10.1007/s10048-013-0362-0 23595507

[B35] Ricci C. Riolo G. Battistini S. (2021). Molecular genetic analysis of cerebral cavernous malformations: an update. Vessel Plus 5, 31. 10.20517/2574-1209.2021.28 PMC8005105 33810005

[B36] Richards S. Aziz N. Bale S. Bick D. Das S. Gastier-Foster J. (2015). Standards and guidelines for the interpretation of sequence variants: a joint consensus recommendation of the American College of medical genetics and genomics and the association for molecular Pathology. Genet. Med. 17 (5), 405–424. 10.1038/gim.2015.30 25741868 PMC4544753

[B37] Robinson J. T. Thorvaldsdóttir H. Winckler W. Guttman M. Lander E. S. Getz G. (2011). Integrative genomics viewer. Nat. Biotechnol. 29 (1), 24–26. 10.1038/nbt.1754 21221095 PMC3346182

[B38] Rossi A. Kontarakis Z. Gerri C. Nolte H. Hölper S. Krüger M. (2015). Genetic compensation induced by deleterious mutations but not gene knockdowns. Nature 524 (7564), 230–233. 10.1038/nature14580 26168398

[B39] Sahoo T. Johnson E. W. Thomas J. W. Kuehl P. M. Jones T. L. Dokken C. G. (1999). Mutations in the gene encoding KRIT1, a Krev-1/rap1a binding protein, cause cerebral cavernous malformations (*CCM1*). Hum. Mol. Genet. 8 (12), 2325–2333. 10.1093/hmg/8.12.2325 10545614

[B40] Schwefel K. Spiegler S. Much C. D. Felbor U. Rath M. (2020). CRISPR/Cas9-mediated generation of human endothelial cell knockout models of CCM disease. Methods Mol. Biol. 2152, 169–177. 10.1007/978-1-0716-0640-7_13 32524552

[B41] Spiegler S. Najm J. Liu J. Gkalympoudis S. Schröder W. Borck G. (2014). High mutation detection rates in cerebral cavernous malformation upon stringent inclusion criteria: One-third of probands are minors. Mol. Genet. Genomic Med. 2 (2), 176–185. 10.1002/mgg3.60 24689081 PMC3960060

[B42] Spiegler S. Rath M. Hoffjan S. Dammann P. Sure U. Pagenstecher A. (2018a). First large genomic inversion in familial cerebral cavernous malformation identified by whole genome sequencing. Neurogenetics 19 (1), 55–59. 10.1007/s10048-017-0531-7 29197946

[B43] Spiegler S. Rath M. Paperlein C. Felbor U. (2018b). Cerebral cavernous malformations: An update on prevalence, molecular genetic analyses, and genetic counselling. Mol. Syndromol. 9 (2), 60–69. 10.1159/000486292 29593473 PMC5836221

[B44] Whiffin N. Karczewski K. J. Zhang X. Chothani S. Smith M. J. Evans D. G. (2020). Characterising the loss-of-function impact of 5' untranslated region variants in 15, 708 individuals. Nat. Commun. 11 (1), 2523. 10.1038/s41467-019-10717-9 32461616 PMC7253449

[B45] Wright C. F. Quaife N. M. Ramos-Hernández L. Danecek P. Ferla M. P. Samocha K. E. (2021). Non-coding region variants upstream of *MEF2C* cause severe developmental disorder through three distinct loss-of-function mechanisms. Am. J. Hum. Genet. 108 (6), 1083–1094. 10.1016/j.ajhg.2021.04.025 34022131 PMC8206381

[B46] Wüstehube J. Bartol A. Liebler S. S. Brütsch R. Zhu Y. Felbor U. (2010). Cerebral cavernous malformation protein CCM1 inhibits sprouting angiogenesis by activating DELTA-NOTCH signaling. Proc. Natl. Acad. Sci. U. S. A. 107 (28), 12640–12645. 10.1073/pnas.1000132107 20616044 PMC2906569

[B47] Zhang F. Lupski J. R. (2015). Non-coding genetic variants in human disease. Hum. Mol. Genet. 24 (R1), R102–R110. 10.1093/hmg/ddv259 26152199 PMC4572001

[B48] Zhou Z. Tang A. T. Wong W. Y. Bamezai S. Goddard L. M. Shenkar R. (2016). Cerebral cavernous malformations arise from endothelial gain of MEKK3-KLF2/4 signalling. Nature 532 (7597), 122–126. 10.1038/nature17178 27027284 PMC4864035

