# Supplementary material for: Using CRISPR/Cas9 genome editing in human iPSCs for deciphering the pathogenicity of a novel CCM1 transcription start site deletion
Source: Front Mol Biosci. 2022 Aug 25;9:953048. doi: 10.3389/fmolb.2022.953048 (PMC9453596; doi:10.3389/fmolb.2022.953048)
Supplement: Supplementary file 1 [file DataSheet1.PDF]

Supplementary Material

1 Supplementary Figures

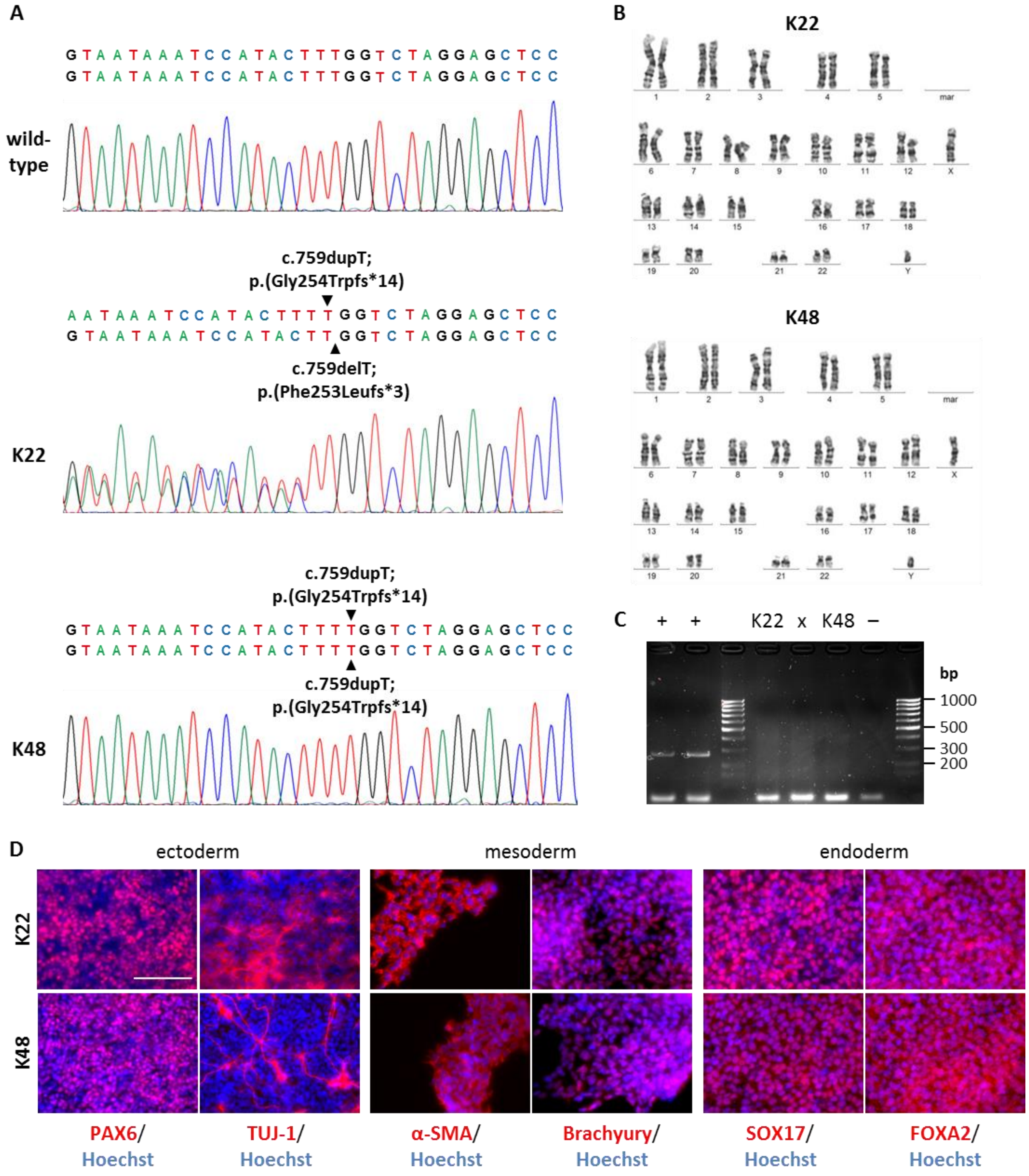

Supplementary Figure 1. Quality control analyses for AICS-0023-derived *CCM1*$^{-/-}$ iPSC clones. **(A)** Sanger sequences showing the CRISPR/Cas9-induced variants. **(B)** Karyotyping by GTG staining. A composite karyotype from 20 metaphase spreads is given. **(C)** PCR test for mycoplasma contamination (mycoplasma-specific band: 270 bp; + = positive controls, – = negative control; x = clone not included in this article). **(D)** Immunofluorescence analyses for ectoderm markers PAX6 and TUJ-1, mesoderm markers α-SMA and Brachyury, and endoderm markers SOX17 and FOXA2 after differentiation into the three germ layers (scale = 100 µm).

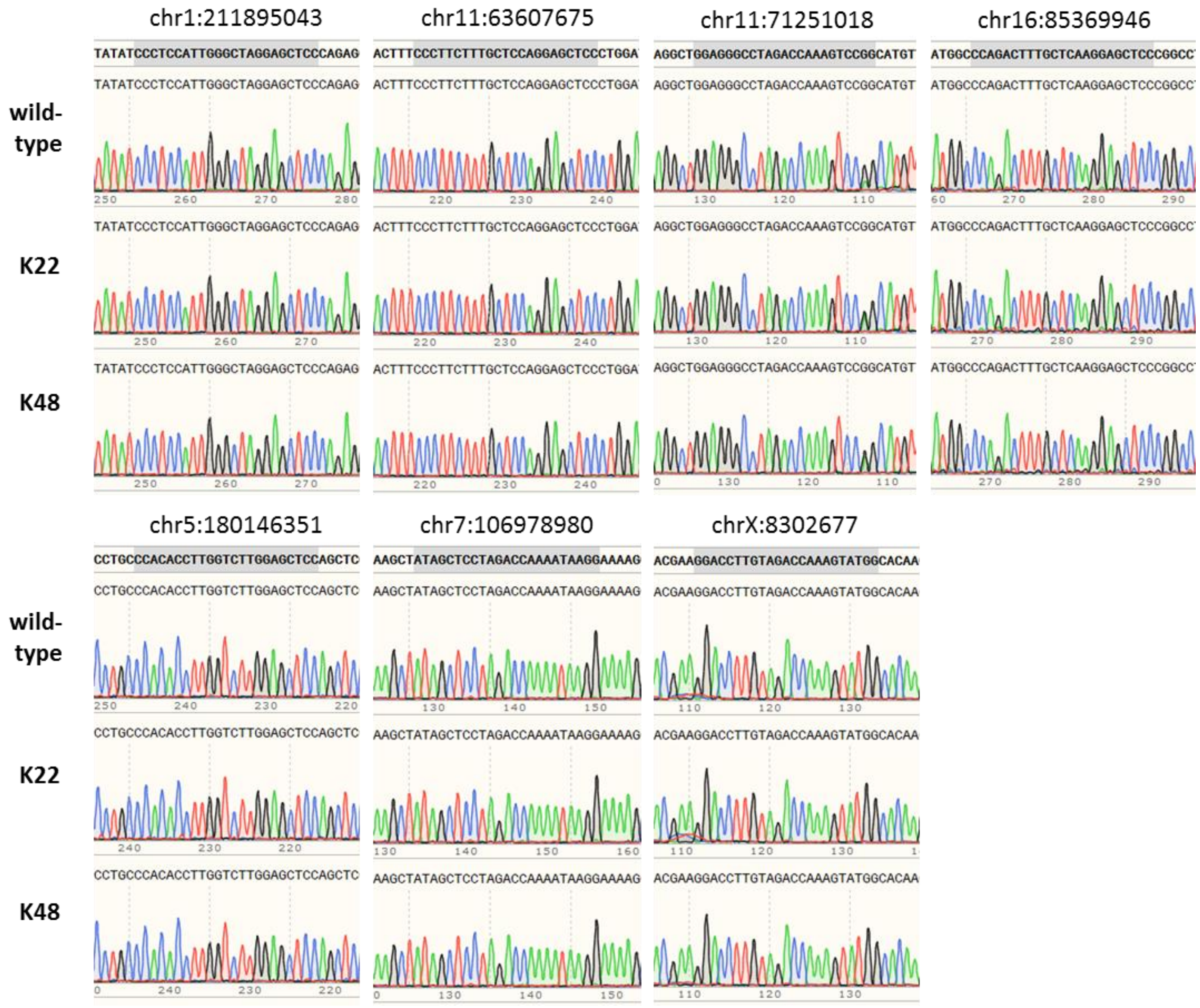

Supplementary Figure 2. Sanger sequencing of seven top off-target sites for *CCM1*$^{-/-}$ AICS-0023 iPSCs. Potential off-target sites are highlighted in grey.

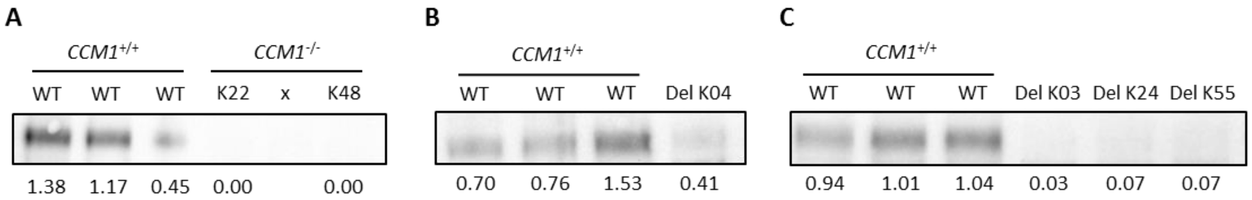

Supplementary Figure 3. Independently performed western blot analyses for *CCM1*$^{-/-}$ AICS-0023 iPSCs **(A)**, for HEK293T clone Del K04 **(B)**, and for *CCM1*$^{del/del}$ AICS-0023 iPSCs **(C)**. Total protein served as reliable loading control (Tin et al., 2018). Expression levels normalized to the wild-type (WT) control group are given. x = clone not included in this article. Reference: Tin, A., Li, Y., Brody, J.A., Nutile, T., Chu, A.Y., Huffman, J.E., et al. (2018). Large-scale whole-exome sequencing association studies identify rare functional variants influencing serum urate levels. *Nat Commun* 9(1)**,** 4228. doi: 10.1038/s41467-018-06620-4.

2 Supplementary Tables

Supplementary Table 1. Oligonucleotides used for CRISPR/Cas9 editing, PCR and quantitative PCR analyses.

analysis	target	sequence (5'-3') / assay
PCR/sequencing	*CCM1*<br>(Cerebral Cavernous Malformations 1)	for: CCACTGTCGTTCCCTAATCA<br>rev: TCGGGTAAGAGTTGAATGAA
PCR/sequencing for deletion region	*CCM1*	for: CAGGCGGAGGCTGATGTG<br>rev: CCTCCTGTTTCCCGAAGGAC
crRNA guide sequence<br>(combination i)	*CCM1*	GCGCGGGGGAACCCTCAAGA/<br>ATGAGCCGGGCTTGACCGCG
crRNA guide sequence<br>(combination ii)	*CCM1*	GCGCGGGGGAACCCTCAAGA/<br>AGGCGGCGCAGCGGCCGGAG
crRNA guide sequence<br>(combination iii)	*CCM1*	GCGCGGGGGAACCCTCAAGA/<br>CGGCGAGGCTCAGACTGGGT
sgRNA guide sequence	*CCM1*	GGAGCTCCTAGACCAAAGTA
qPCR	*RPLP0*<br>(Ribosomal Protein Lateral Stalk Subunit P0)	for: TCGACAATGGCAGCATCTAC<br>rev: ATCCGTCTCCACAGACAAGG
qPCR	*CCM1*	Hs.PT.56a.600880 (Integrated DNA Technologies)
qPCR	*ANKIB1*<br>(Ankyrin Repeat And IBR Domain Containing 1)	Hs.PT.58.27551849 (Integrated DNA Technologies)

qPCR	*KLF2*<br><br>(Kruppel Like Factor 2)	Hs.PT.58.39339409 (Integrated DNA Technologies)
qPCR	*KLF4*<br><br>(Kruppel Like Factor 4)	Hs.PT.58.45542593 (Integrated DNA Technologies)
qPCR	*THBS1*<br><br>(Thrombospondin 1)	Hs.PT.58.45510824 (Integrated DNA Technologies)
qPCR	*NOS3*<br><br>(Nitric Oxide Synthase 3)	Hs.PT.58.21447620 (Integrated DNA Technologies)
qPCR	*HEY2*<br><br>(Hes Related Family BHLH Transcription Factor With YRPW Motif 2)	Hs.PT.58.582440 (Integrated DNA Technologies)
Mycoplasma Test		for: GGGAGCAAACAGGATTAGATACCCT<br><br>rev: TGCACCATCTGTCACTCTGTTAACCTC
PCR/sequencing for off-target sites	chr1:211895043 (GRCh37/hg19)	for: GCTCTGCAGATTTCTGGGGT<br><br>rev: GAAACAGCGGACTGAAGGGA
PCR/sequencing for off-target sites	chr11:63607675 (GRCh37/hg19)	for: TCCACCTCTCCCTTCGTCTC<br><br>rev: ATTGGAACCCCAAACGCAAC
PCR/sequencing for off-target sites	chr11:71251018 (GRCh37/hg19)	for: TGGGCTTCTCTCCCCTACAA<br><br>rev: TGCCATCCTGTGAGAAGGTG
PCR/sequencing for off-target sites	chr16:85369946 (GRCh37/hg19)	for: GAGATATGGAGGAACAGGGCG<br><br>rev: ATCCTGCACCTCTGATTCTGC

PCR/sequencing for off-target sites	chr5:180146351 (GRCh37/hg19)	for: TATGGGTGGTCCCCAATCCA<br>rev: AGGTGAAAACAACGGCCAGA
PCR/sequencing for off-target sites	chr7:106978980 (GRCh37/hg19)	for: ACCAAGGCTACAAAATGGGGA<br>rev: CTCTCTGTTGTTTCCTCTACTGC
PCR/sequencing for off-target sites	chrX:8302677 (GRCh37/hg19)	for: TGTCAGCATGGGCTTTACCAA<br>rev: GACCAGTATTGCGTCCCGTG

Supplementary Table 2. Antibodies used in immunofluorescence and western blot analyses.

**antibody**<br>**(target)**	**clone/catalog number, RRID**	**vendor**	**dilution**
primary antibodies			
Mouse Anti-SSEA4<br>(Stage-Specific Embryonic Antigen 4)	A24866, RRID:AB_2651001	Thermo Fisher Scientific	1:100
Rabbit Anti-OCT4<br>(Octamer-Binding Transcription Factor 4)	A24867, RRID:AB_2650999	Thermo Fisher Scientific	1:200
Rat Anti-SOX2<br>(SRY-Box Transcription Factor 2)	A24759, RRID:AB_2651000	Thermo Fisher Scientific	1:100
Mouse Anti-TRA-1-60<br>(T Cell Receptor Alpha Locus)	A24868, RRID:AB_2651002	Thermo Fisher Scientific	1:100
Mouse Anti-Human PAX6<br>(Paired Box 6)	561462, RRID:AB_10715442	BD Biosciences	1:200

Mouse Anti-Beta-III Tubulin<br><br>(Tubulin Beta 3 Class III)	MAB1195, RRID:AB_357520	R&D Systems	1:100
Goat Anti-Human/Mouse Brachyury<br><br>(T-Box Transcription Factor T)	AF2085, RRID:AB_2200235	R&D Systems	1:20
Mouse Anti-Alpha Smooth Muscle Actin [1A4]<br><br>(Actin Alpha 2, Smooth Muscle)	ab7817, RRID:AB_262054	Abcam	1:200
Goat Anti-Human SOX17<br><br>(SRY-Box Transcription Factor 17)	AF1924, RRID:AB_355060	R&D Systems	1:20
Goat Anti-Human HNF-3 beta/FoxA2<br><br>(Forkhead Box A2)	AF2400, RRID:AB_2294104	R&D Systems	1:20
Mouse Anti-CD31 (PECAM-1) (89C2)<br><br>(Platelet And Endothelial Cell Adhesion Molecule 1)	3528, RRID:AB_2160882	Cell Signaling	1:800
Rabbit Anti-VE-Cadherin (D87F2) XP<br><br>(Cadherin 5)	2500, RRID:AB_10839118	Cell Signaling	1:400

Rabbit Anti-GAPDH<br>(Glyceraldehyde-3-Phosphate Dehydrogenase)	PA1-16777, RRID:AB_568552	Thermo Fisher Scientific	1:500
Rabbit Anti-KRIT1 [EPR16560]<br>(Krev Interaction Trapped Protein 1)	ab196025	Abcam	1:200
secondary antibodies			
Alexa Fluor 555 Goat Anti-Mouse IgG H+L	ab150114, RRID:AB_2687594	Abcam	1:500 / 1:200
Alexa Fluor 555 Donkey Anti-Rabbit	A24869, RRID:AB_2651006	Thermo Fisher Scientific	1:250
Alexa Fluor 555 Goat Anti-Rat IgG H+L	A-21434, RRID:AB_2535855	Thermo Fisher Scientific	1:500
Alexa Fluor 555 Goat Anti-Mouse IgM	A24871, RRID:AB_2651009	Thermo Fisher Scientific	1:250
Alexa Fluor 555 Donkey Anti-Goat IgG H+L	ab150130, RRID:AB_10894526	Abcam	1:200
Alexa Fluor 555 Goat Anti-Rabbit IgG H+L	A-21429, RRID:AB_2535850	Thermo Fisher Scientific	1:500
Goat Anti-Rabbit IgG H+L (HRP)	ab205718, RRID:AB_2819160	Abcam	1:400
